# Updating in working memory predicts greater emotion reactivity to and facilitated recovery from negative emotion-eliciting stimuli

**DOI:** 10.3389/fpsyg.2015.00372

**Published:** 2015-04-09

**Authors:** Madeline L. Pe, Peter Koval, Marlies Houben, Yasemin Erbas, Dominique Champagne, Peter Kuppens

**Affiliations:** ^1^Research Group of Quantitative Psychology and Individual Differences, Faculty of Psychology and Educational Sciences, KU LeuvenLeuven, Belgium; ^2^School of Psychology, Faculty of Health Sciences, Australian Catholic UniversityMelbourne, VIC, Australia

**Keywords:** updating, working memory, emotion reactivity, emotion recovery, emotion responding

## Abstract

That emotions change in response to emotion-eliciting events is a natural part of human life. However, it is equally important for emotions to return to baseline once the emotion-eliciting events have passed. This suggests that the ability to emotionally react to and recover from emotion-eliciting events is critical for healthy psychological functioning. But why do individuals differ in their emotion reactivity and recovery? The present work postulates that the ability to update emotional information in working memory (WM) may explain individual differences in emotion reactivity and recovery. Two studies are presented, which examined whether updating ability was related to emotion reactivity and recovery. In Study 1, we assessed participants' self-reported affect as they viewed negative and positive films. Our results revealed that better updating ability was related to greater emotion reactivity and facilitated (i.e., quicker) recovery from watching negative films. In Study 2, participants recalled a recent angering event, and were then instructed to either ruminate about or reappraise the event. Results revealed that updating ability was again related to greater emotion reactivity and facilitated (i.e., successful) emotion recovery in response to the angering event, and that this was unrelated to the emotion regulation strategy used. These findings identify the ability to update emotional information in WM as a possible mechanism in emotion responding.

## Introduction

Suppose another driver cuts you off, making you feel upset. Why do you feel this way? According to emotion theories, one of the more critical functions of emotions is to organize and motivate rapid actions in order to adaptively respond to immediate threats to survival or well-being (Izard, 2009). In other words, it is adaptive for emotions to change in response to changing situational demands since they prompt the organism to immediately act or to prepare for action. However, once alerted, it is equally important for the organism to regulate the initial emotional response back to baseline to avoid overload or disruption by sustained emotional arousal (Block and Kremen, 1996). Therefore, the ability to emotionally react to and recover from an emotion-eliciting event is critical for healthy psychological functioning. Indeed, the ability to modify emotional responses in accordance with the situation has been implicated as an important ingredient of psychological health (Kashdan and Rottenberg, 2010); it is also particularly apparent in the fact that when they become resistant to change, they are associated with emotion disorders like depression (Bylsma et al., 2008; Aldao et al., 2010; Kuppens et al., 2010; Pe et al., 2015).

But why do individuals differ in how much they react to and recover from an emotion-eliciting event? In this paper, we propose that one process involved in modulating emotion reactivity and recovery is updating—a specific executive function closely related to the construct of working memory (WM; Schmiedek et al., 2009; Hofmann et al., 2012; Wilhelm et al., 2013). Updating refers to the ability to modify the contents of WM to accommodate incoming relevant information (Morris and Jones, 1990; Miyake et al., 2000); the ability to emotionally react to and recover from an emotion-eliciting event should at least partly rely on the ability to update information in WM—that is, the ability to change the contents in WM to accommodate new information as it becomes available.

Going back to the earlier example: Suppose a driver cuts you off. Your updating ability allows you to modify your thought content based on the current situational demand (e.g., from enjoying the music in your car to experiencing a dangerous situation). The activation of negative thoughts is associated with your experience of increased negative emotions (Joormann, 2010). However, once the negative event has passed, it becomes critical to again update the contents of your WM with the information relevant to the new situation (e.g., you are safely driving again). Successfully updating the contents of your WM to fit the new situation would result in decreased negative emotions since the focus of your attention changes from the negative event to the new event. Conversely, poor updating would lead to increased interference of the previous thought content to the current thought content, resulting in a blunted emotional responding to the new situation.

### Updating, emotion reactivity, and emotion recovery

Emotion reactivity and emotion recovery are processes that involve emotion regulation. Emotion regulation refers to the process by which individuals influence which emotions they have, when they have them, and how they experience and express these emotions (Gross, 1998). For example, people may try to modulate the intensity of their emotional response to appropriately fit the emotional event. Similarly, once the emotional event has passed, individuals would regulate their emotions again to return their heightened emotional response back to baseline.

There is a growing number of research demonstrating that updating in WM is associated with increased ability to regulate emotions (Hofmann et al., 2012). For instance, individuals with better ability to update their WM felt less disgust when they were instructed to appraise a disgusting stimulus in an unemotional manner, and they also experienced less negative emotions when instructed to actively decrease their negative emotions in response to negative pictures (Schmeichel et al., 2008; McRae et al., 2012). Moreover, individuals with better updating ability were also able to successfully regulate emotions even when they were not instructed to do so. For example, given a negative feedback, they spontaneously regulated their emotions through self-enhancement (Schmeichel and Demaree, 2010). Furthermore, their ability to successfully regulate their emotions was also evident in their self-reported daily life experiences. In a 7-day experience sampling study, individuals with better updating ability experienced a greater decrease and increase in their negative and positive emotions (respectively) after they have reported the use of reappraisal as an emotion regulation strategy (Pe et al., 2013b).

### The present study

To investigate whether individuals with higher updating ability would exhibit both increased emotion reactivity and facilitated recovery, we designed two studies to have an emotion-eliciting event as well as a baseline (or a rest period) before and after the emotion-eliciting event. The change in emotional response from baseline to the emotion-eliciting event measures reactivity to the event, while the change in emotional response from the emotion-eliciting event to the subsequent rest period measures recovery from the event. To measure updating ability in both studies, we utilized an emotional variant of the n-back task, the emotional 2-back (Pe et al., 2013a,b). The underlying rationale is that an updating task that specifically involves valenced emotional information would be particularly relevant for picking up the processes operating during emotion responding. We predicted that individuals with high updating ability would have greater emotion reactivity to and facilitated recovery from an emotion-eliciting event.

## Study 1

Our goal for the first study was to empirically test whether a relationship between updating ability and emotion reactivity and recovery exists. Here, we wanted to explore emotion reactivity to and recovery from both positive and negative emotion-eliciting stimuli, and examine whether updating ability would be related to emotion responding to both positive and negative emotion-eliciting stimuli.

Participants watched a series of movie clips that were of positive, neutral, or negative valence. After they had watched a clip, participants were given a brief rest period. Participants rated their emotional responses after each film-clip and rest period. The presence of rest periods before and after each film-clip allowed the measurement of reactivity to and recovery from the emotion-eliciting stimuli. We hypothesize that high updating ability would be related to greater reactivity to and facilitated recovery from watching both negative and positive film-clips.

### Materials and methods

#### Participants

We aimed to recruit 200 students, who were just commencing their first year of tertiary education at a Belgian university or higher education institute. To ensure our participants represented a broad range of psychological well-being levels, we employed a stratified sampling approach (Ingram and Siegle, 2009). From an initial pool of 686 students commencing their first year of tertiary education, we selected 180 participants who reflected a broad range of pre-screening scores on the Center for Epidemiologic Studies Depression Scale (CES-D; Radloff, 1977; Range = 0–39; *M* = 14.41, *SD* = 8.41). To achieve the goal of 200 participants, we recruited an additional 22 participants after the study had already begun and therefore did not complete the CES-D pre-screening. This gave us a final sample of 202 participants (111 female) ranging in age from 17 to 24 years (*M* = 18.33; *SD* = 0.95). We again measured the CES-D scores of the participants at the start of the study. The mean CES-D score for the 22 participants who were not pre-screened (*M* = 11.86, *SD* = 7.11) did not differ significantly from the mean CES-D score of the initial 180 participants who completed the pre-screening (*M* = 12.55; *SD* = 7.80), *t*_(200)_ = −0.393, *p* = 0.695. Participants were paid a maximum of 60 euros for their participation.

#### Procedure

The present study was part of a larger 7-day experience sampling study on emotions. The emotional n-back task was administered on the first day of the experience sampling study (Day 1), whereas the film-clip task was administered 1 day after the experience sampling study ended (Day 8). Both the emotional n-back and film-clip tasks were programmed in E-prime 2.0 (Schneider et al., 2002) and administered in individual cubicles. This study was approved by the local ethics committee of the Faculty of Psychology and Educational Sciences, KU Leuven, Belgium. Written informed consent was obtained from all participants.

#### Materials

##### Updating task

Participants completed the emotional 2-back task (Pe et al., 2013a,b), a modified version of the n-back, a classic updating task. Instead of using non-emotional stimuli (e.g., letters), the emotional 2-back uses emotional words as stimuli, and participants have to decide whether the valence of the current word (trial *n*) matches the valence of the word presented 2 trials back (trial *n*-2). This task measures updating emotional information in WM by requiring participants, at every trial (trial *n*), to remove previously relevant emotional information from WM, information that has now become irrelevant (trial *n*-3), encode newer relevant information in WM (trial *n*) and match the valence of this new information with relevant, but old information in WM (trial *n*-2).

A total of 47 positive and 49 negative words were selected from the Affective Norms of English Words list (Bradley and Lang, 1999) and translated into Dutch. Words were identified as negative and positive if their valence ratings ranged from 1 to 4 and 6 to 9, respectively, and they were also matched in word length, number of syllables, and arousal levels (see Pe et al., 2013b). The task consisted of 24 practice trials (not scored) and 96 actual trials separated into four blocks of 24 trials. The first two trials of every block cannot be scored (as there are no stimuli two trials before each of the first two trials), leaving a total of 88 relevant trials for analysis. In each trial, participants viewed a single affective word presented centrally for 500 ms followed by a 2500 ms intertrial interval. Participants were instructed to indicate whether the valence of the current word (i.e., newer, incoming stimulus, trial *n*) had the same (match) or different (non-match) valence as the word two trials back (trial *n*-2) by pressing the “1” or “2” key, respectively.

There were 44 match trials (22 trials were positive-valenced stimuli, i.e., the current stimulus, trial *n*, and the stimulus two trials back, trial *n*-2, were both positive) and 44 non-match trials (21 trials were positive-valenced stimuli; i.e., trial *n* was positive, but trial *n*-2 was negative). Non-responses or omissions were counted as errors. To measure participants' ability to update emotional information, we calculated the mean accuracy scores across all trials (see also Pe et al., 2013a,b) (KR_20_ = 0.84).

##### Film task

The film-task is an adapted version of the film-task developed by Koval et al. (2013). Participants first read general instructions and then completed a practice trial of responding to the emotion items for the film task. In the practice trials, participants watched a neutral film and practiced responding to each of the emotion items within the specified time limit (i.e., a maximum of 5 s). Next, participants completed a baseline rating of their subjective experiences of positive (happy, relaxed, excited) and negative (sad, anxious, angry, depressed) emotions. After, participants viewed 10 emotional film-clips (four negative, four positive, and two neutral films) in a fixed order and provided ratings of their positive and negative emotions. Film clips were selected from a validated database of 70 emotion-eliciting film excerpts based on their positive- and negative-valence eliciting norm scores (Schaefer et al., 2010), and were shown in the following order: Trainspotting[1] (negative), Schindler's List[3] (negative), Blue[2] (neutral), Trainspotting[3] (positive), Blue[3] (neutral), The Dentist (negative), Benny and Joon (positive), There is Something About Mary[1] (positive), Indiana Jones and the Last Crusade (negative), When a Man Loves a Woman (positive). These film clips were based on emotion ratings from Schaefer et al.'s (2010) validation study. To avoid ceiling effects, the selected film clips were not among the 20 highest on “emotional arousal” in Schaefer et al.'s (2010) validation study. To limit the overall length of the task, the selected clips were shorter than 3 min (Range 0:25–2:28 min). In addition, only English-speaking film clips with Dutch subtitles were used.

Moreover, there was a 30 s “rest period” following each film-clip, during which participants were asked to keep their attention on the screen, and after which they also rated their current positive and negative emotions. Emotion items were rated on a scale from 0 (not at all) to 6 (very much).

Data from the practice trial were not analyzed, leaving 21 emotion measurements per participant (i.e., one baseline measure, one measure after each of the 10 film-clips, and one measure after each of the 10 rest periods). To ensure that only meaningful responses were included in our final analyses, we removed emotion ratings with very fast response times (RTs). Specifically, emotion ratings with RTs below 300 ms were replaced with missing values, as were emotion ratings with RTs more than 3 SDs below each participant's mean RT for each specific emotion item. This affected 88 out of 25452 emotion ratings (0.35%). For each of the 21 measurement occasions, we averaged ratings on the four negative items to form a negative emotionality (NE) scale (between-subject reliability = 0.90), and we averaged ratings on the three positive items to form a positive emotion (PE) scale (between-subject reliability = 0.92).

#### Statistical model

Our main goal was to examine whether updating ability was related to emotion reactivity and recovery. To address this aim, we utilized piecewise multilevel regression, which allowed us to take into account the nested structure of the data (i.e., measurement occasions were nested within participants) and the non-linearity of the model (Naumova et al., 2001). This model also allowed us to simultaneously estimate the reactivity and recovery slopes.

We modeled emotion reactivity to and emotion recovery from the film-clips by averaging PE and NE scores across all measurement occasions (a) during the baseline and/or after the rest period before a film-clip was shown (baseline phase), (b) right after watching a film-clip (event phase), and (c) following the rest period after watching a film-clip (rest phase). We calculated three such aggregated scores separately for negative and positive film-clips, which we used for all subsequent analyses. For instance, for negative film clips, the PE or NE scores for the baseline phase (a) was the average of the “rest” PE or NE scores of the following films: Schindler's list [3], Blue [3], There is Something About Mary[1], and the baseline emotion scores right before Trainspotting[1]. The PE or NE scores for the event phase (b) was the average PE or NE scores of the following films: Trainspotting[1], Schindler's List[3], The Dentist, and Indiana Jones and the Last Crusade. The PE or NE scores for the rest phase (c) was the average PE or NE scores of the “rest” scores of the following films: Trainspotting[1], Schindler's List[3], The Dentist, and Indiana Jones and the Last Crusade. Since we were only interested in emotional-eliciting events, we excluded the neutral film-clips from the main analyses.

For each film-clip valence, we created a phase variable (*phase*_*ij*_) in which measurement occasions during the different phases were recoded into numerical form. Specifically, measurement occasion during baseline phase was coded as 1, the measurement occasion during the event phase was coded as 2, and the measurement occasion during the rest phase was coded as 3.

Following the procedure of Naumova et al. (2001), we created a variable *time*_*ij*_, which was the time of measurement of the phase variable relative to the emotion-eliciting event, *time*_*ij*_ = *phase*_*ij*_ − *event*_*i*_, in which *event*_*i*_ was the event phase for subject *i*. For example, the baseline phase was recoded to *time_*ij*_* = −1 because the event (i.e., the event phase, initially coded as 2) was subtracted from the baseline phase (initially coded as 1). A similar procedure was done for the event and rest phases, yielding the recoded values of 0 and 1, respectively. We let *event*_*i*_ be an indicator: β_*ij*_ = 1 for the time period before the event, *time*_*ij*_ < 0, and β_*ij*_ = 0 for the time period after the event, *time*_*ij*_ ≥ 0. This resulted in a combined model at Level 1:

$$NE_{ij}=\delta_{0i}\;+\;\delta_{1i}time_{ij}(\beta_{ij})\;+\;\delta_{2i}time_{ij}(1-\beta_{ij})\;+\;e_{ij}$$

where δ_0*i*_ is an intercept, reflecting subject *i*'s level of NE experienced after watching the movie clip, δ_1*i*_ is subject *i*'s reactivity slope (reflecting change in NE from baseline to film), and δ_2*i*_ is subject *i*'s recovery slope (reflecting change in NE from film to rest), and *e*_*ij*_ is the measurement error at the *j*th occasion for the *i*th subject.

Since we are interested in the relationship between updating ability and the reactivity and recovery slopes, we included updating ability *updating*_*i*_ (standardized) as a Level 2 predictor for each of the Level 1 parameters, as shown below:

$$\begin{array}{l} \delta_{0i}=\gamma_{00}+\gamma_{01}(updating_{i})\;+\;r_{0i}\\ \delta_{1i}=\gamma_{10}+\gamma_{11}(updating_{i})\;+\;r_{1i}\\ \delta_{2i}=\gamma_{20}+\gamma_{21}(updating_{i})\;+\;r_{2i} \end{array}$$

At Level 2, γ_00_ reflects the NE score after watching a negative film for participants with average updating ability, whereas γ_01_ reflects how individual differences in NE scores after watching a negative film are a function of individual differences in updating ability. The parameters γ_10_ and γ_20_ reflect NE reactivity and recovery slopes for participants with average updating ability, respectively. Finally, of particular interest to our research question, γ_11_ and γ_21_ reflect the extent to which individual differences in updating ability are related to individual differences in NE reactivity and recovery slopes, respectively.

We did this analysis four times, examining participants' PE and NE responses to both positive and negative film-clips. All piecewise multilevel regression analyses were conducted using HLM 7.01 (Raudenbush et al., 2013).

### Results

We present the descriptive statistics of the all variables used in Study 1 in Table 1.

**Table 1:** **Descriptive statistics of the variables in Study 1 (*n* = 202)**.

		***M***	***SD***	**Actual range**	**Possible range**	**Correlations**											
						**1**	**2**	**3**	**4**	**5**	**6**	**7**	**8**	**9**	**10**	**11**	**12**
1	Emotional n-back	0.64	0.12	0.25–0.92	0.00–1.00	–											
**NEGATIVE FILMS**																	
2	NE (baseline)	0.60	0.62	0.00–3.60	0.00–6.00	−0.09	–										
3	NE (film)	1.93	1.06	0.13–5.06	0.00–6.00	0.05	**0.67**	–									
4	NE (rest)	1.05	0.92	0.00–4.25	0.00–6.00	−0.06	**0.82**	**0.82**	–								
5	PE (baseline)	3.29	0.95	0.00–5.75	0.00–6.00	0.01	**−0.28**	−0.11	**−0.25**	−							
6	PE (film)	1.62	1.01	0.00–4.83	0.00–6.00	0.02	**−0.21**	**−0.48**	**−0.40**	**0.56**	–						
7	PE (rest)	2.54	1.06	0.00–5.42	0.00–6.00	0.06	**−0.32**	**−0.34**	**−0.51**	**0.75**	**0.73**	–					
**POSITIVE FILMS**																	
8	NE (baseline)	0.57	0.65	0.00–3.25	0.00–6.00	−0.02	**0.84**	**0.72**	**0.89**	**−0.23**	**−0.24**	**−0.34**	–				
9	NE (film)	0.39	0.50	0.00–3.13	0.00–6.00	0.03	**0.77**	**0.62**	**0.66**	**−0.19**	**−0.16**	**−0.16**	**0.79**	–			
10	NE (rest)	0.34	0.53	0.00–3.13	0.00–6.00	−0.04	**0.87**	**0.51**	**0.65**	**−0.22**	−0.08	**−0.16**	**0.82**	**0.89**	–		
11	PE (baseline)	3.00	0.98	0.00–6.00	0.00–6.00	0.10	**−0.28**	**−0.17**	**−0.34**	**0.79**	**0.59**	**0.84**	**−0.30**	**−0.19**	**−0.22**	–	
12	PE (film)	3.66	0.98	0.50–6.00	0.00–6.00	0.02	**−0.25**	−0.13	**−0.26**	**0.68**	**0.48**	**0.59**	**−0.24**	**−0.27**	**−0.25**	**0.76**	–
13	PE (rest)	3.47	1.01	0.00–5.92	0.00–6.00	0.09	**−0.23**	−0.05	**−0.18**	**0.83**	**0.46**	**0.65**	**−0.21**	**−0.21**	**−0.24**	**0.88**	**0.82**

*NE, negative emotions; PE, positive emotions; Figures in bold are significant at p < 0.05*.

#### Reactivity and recovery to film clips

##### Emotion reactivity

As shown in Table 2, results from the piecewise multilevel regression analyses showed that after watching a negative film-clip, participants, on average, experienced significant increases in NE and significant decreases in PE relative to their baseline levels. For positive films, participants, on average, experienced a significant decrease in NE and a significant increase in PE after watching a positive film-clip. The change of their emotions from baseline to the emotion-eliciting event (i.e., watching of film-clips) suggests that participants, on average, exhibited emotion reactivity to these clips (see γ_10_ estimates in Table 2).

**Table 2:** **Moderating effect of updating on emotion reactivity to and emotion recovery from valenced film-clips in Study 1**.

	**Negative emotions**			***R*^2^**	**Positive emotions**			***R*^2^**
	**Coef**	***SE***	**95% CI**		**Coef**	***SE***	**95% CI**	
**NEGATIVE FILMS**								
**Watching the clip**								
Intercept (γ_00_)	1.93^**^	0.07	(1.78, 2.07)		1.62^**^	0.07	(1.48, 1.76)	
Updating (γ_01_)	0.05	0.09	(−0.12, 0.22)	−0.00	0.01	0.07	(−0.13, 0.15)	−0.01
**Reactivity**								
Intercept (γ_10_)	1.33^**^	0.06	(1.22, 1.44)		−1.67^**^	0.06	(−1.79, −1.54)	
Updating (γ_11_)	0.11^*^	0.05	(0.00, 0.21)	0.03	0.04	0.07	(−0.11, 0.18)	−0.01
**Recovery**								
Intercept (γ_20_)	−0.88^**^	0.04	(−0.96, −0.79)		0.92^**^	0.05	(0.82, 1.01)	
Updating (γ_21_)	−0.11^*^	0.04	(−0.19, −0.02)	0.07	0.02	0.05	(−0.08, 0.12)	−0.01
**POSITIVE FILMS**								
**Watching the clip**								
Intercept (γ_00_)	0.39^**^	0.04	(0.32, 0.46)		3.66^**^	0.07	(3.53, 3.79)	
Updating (γ_01_)	0.02	0.04	(−0.05, 0.09)	−0.01	−0.01	0.08	(−0.15, 0.14)	−0.00
**Reactivity**								
Intercept (γ_00_)	−0.18^**^	0.03	(−0.23, −0.12)		0.67^**^	0.05	(0.57, 0.76)	
Updating (γ_01_)	0.03	0.04	(−0.05, 0.11)	−0.01	−0.06	0.05	(−0.15, 0.03)	0.01
**Recovery**								
Intercept (γ_00_)	−0.06^**^	0.02	(−0.09, −0.02)		−0.19^**^	0.04	(−0.27, −0.11)	
Updating (γ_01_)	−0.04	0.03	(−0.09, 0.02)	0.01	0.06	0.05	(−0.03, 0.15)	0.01

*We use R^2^, the proportion of reduction in variance, to compute for effect sizes, which represents the percent of residual variance that has been “explained” by the added predictor (Nezlek, 2008). This is calculated from (var_nopredictor_−var_predictor_)/var_nopredictor_ (Nezlek, 2012; Peugh, 2010). The “nopredictor” subscript represents the variance estimate from the model prior to adding a predictor, and the “predictor” subscript represents the variance from a model that contains a predictor variable (Peugh, 2010). However, since the issue of calculating effect sizes in a multi-level context is far from resolved (Nezlek, 2008), we request that readers interpret these effect sizes with caution*.

** p < 0.01;

* *p < 0.05*.

##### Emotion recovery

As displayed in Table 2, results showed that following a short rest period after watching negative films, participants, on average, experienced a significant decrease in NE and a significant increase in PE. For positive films, participants, on average, experienced a significant decrease in NE and a significant decrease in PE after the rest period. The change of their emotions from the emotion-eliciting event (i.e., after watching of film-clips) to the rest period for both positive and negative films suggests that participants on average exhibited emotion recovery to these films (see γ_20_ estimates in Table 2).

#### Updating and emotion reactivity and recovery

##### Emotion reactivity

**Figure 1:**
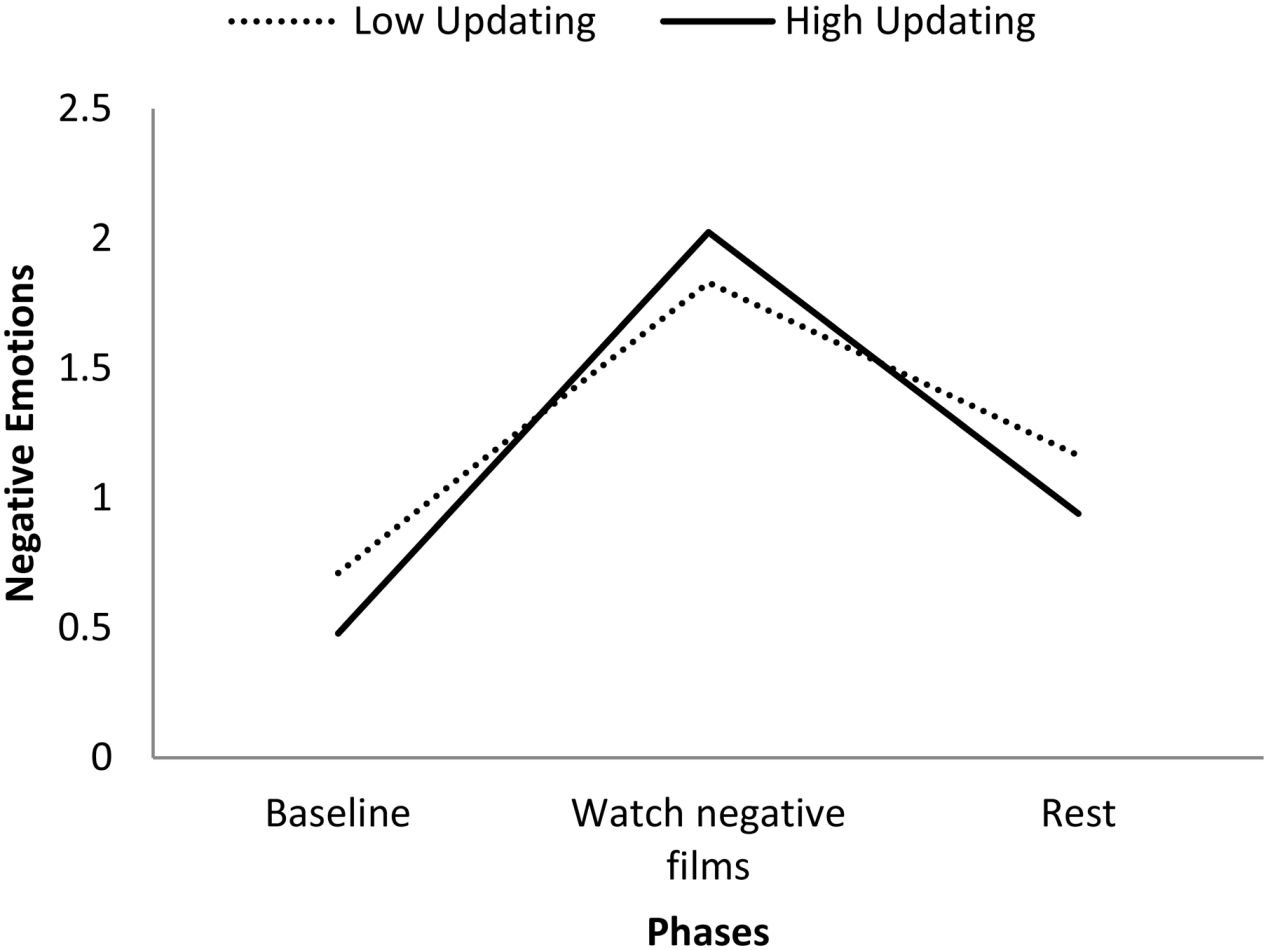
**Negative emotions in Study 1 during the different phases of the negative film-clip task**. Low and high updating represent participants who scored 2SD below and above the sample average emotional updating ability, respectively.

Updating ability was significantly related to NE reactivity to negative films, such that the better the updating ability, the greater the increase in NE after watching negative films (see Table 2 and Figure 1). We applied the simple slope analysis proposed by Preacher et al. (2006) to disentangle this cross-level significant interaction effect. Results revealed that participants with high updating ability (i.e., 2*SD* above the mean) had a greater increase in NE score (β = 1.55, *SE* = 0.13, *p* < 0.001) relative to participants with low updating ability (i.e., 2*SD* below the mean) (β = 1.12, *SE* = 0.11, *p* < 0.001).

No significant relations were found between updating ability and PE reactivity to negative films, or between updating ability and NE or PE reactivity to positive films (see Table 2).

##### Emotion recovery

Similarly, updating ability was significantly related to NE recovery from negative films, such that the better the updating ability, the greater the decrease in NE following the rest period after watching negative films (see Table 2 and Figure 1). We again applied the simple slope analysis proposed by Preacher et al. (2006) to disentangle this cross-level significant interaction effect. Results revealed that participants with high updating ability (i.e., 2*SD* above the mean) had a greater decrease in NE scores (β = −1.09, *SE* = 0.09, *p* < 0.001) relative to participants with low updating ability (i.e., 2*SD* below the mean) (β = −0.67, *SE* = 0.10, *p* < 0.001).

Again, no significant relations were found between updating ability and PE recovery from negative films, or between updating ability and PE or NE recovery from positive films (see Table 2).

##### Other analyses

We also examined whether updating ability would predict differences in positive or negative emotions after the baseline or rest phases for both the negative and positive film-clips. We found no significant correlations between updating ability and emotion levels at the baseline of negative films baseline of positive films, the rest phase of negative films, and the rest phase of positive films (see Table 1).

#### Discussion

In this study, we hypothesized that better updating ability would be related to greater emotion reactivity and facilitated recovery from emotional film-clips. Our findings partially supported this hypothesis, such that the relationship between updating ability and emotion reactivity and recovery was specific to negative emotions in response to negative film-clips. Better updating ability was related to greater NE reactivity to and faster NE recovery from watching negative film-clips. However, this was not the case for PE in response to negative film-clips. Likewise, no association between updating and emotion responding in response to positive film-clips was found.

Our findings in Study 1 suggest that updating may represent a basic ability that plays a part in emotion reactivity and recovery. However, a limitation of the current study is that participants responded to standardized or “objective” emotion-eliciting stimuli. Although this procedure has its merits (i.e., all participants were exposed to the same events), the emotion-eliciting stimuli can be criticized as being “artificial,” and may not reflect emotion responding in response to more real-life emotional events. In the next study, we aimed to remedy this limitation by altering the negative emotion-eliciting event to something more personally relevant to the participants: Participants were asked to recall their most recent unresolved personal angering event. Furthermore, since the results of Study 1 revealed that the relationship of updating ability and emotion reactivity and recovery was specific to NE responding in response to negative stimuli, we focused on emotion reactivity to and recovery from a negative emotion-eliciting stimuli in Study 2.

## Study 2

Our first aim in Study 2 was to replicate and build on the findings of Study 1 using a slightly different paradigm. Our second aim was to sharpen our understanding about the association between updating and emotion regulation, an association that may play an important role in emotion recovery. Previous research has shown that better updating ability was associated with effective use of cognitive emotion regulation strategies, such as reappraisal (Schmeichel et al., 2008; McRae et al., 2012; Pe et al., 2013b) and rumination (Pe et al., 2013b). Specifically, high updating ability was associated with lower negative emotions when reappraisal or rumination was used to regulate emotions. In the current study, we wanted to examine whether we could replicate such findings. To do this, we added an experimental condition, in which participants were given specific instructions to reappraise or to ruminate about the event during the recovery part of the task. Using a paradigm adapted from Ray et al. (2008), we first asked participants to rate their current emotions when they entered the lab (baseline). Then, we instructed participants to recall a recent angering event (recall phase). After, we told participants to either ruminate about or reappraise their angering event (emotion regulation phase). Finally, we asked them to wait for the next instructions (rest phase). Participants rated their emotions after each of these phases. The emotion measures at baseline and recall phase enabled us to measure the participants' reactivity to the angering event, whereas the emotion measures at the recall, emotion regulation and rest phases allowed us to measure the participants' recovery from recalling the angering event. The presence of the emotion regulation manipulation allowed us to further investigate whether updating may play a different role in the recovery from the recall of the angering event during reappraisal vs. rumination.

### Materials and methods

#### Participants

Ninety-three first year University of Leuven psychology students (79 women), whose ages ranged from 17 to 29 years (*M* = 18.51, *SD* = 1.40), participated in this study. Participants earned partial course credit for participation in this study.

#### Procedure

The present study was administered in groups of 15 or less in a room with 25 desktop computers. As participants entered the room, they were randomly assigned to a computer which was programmed to administer either the reappraisal or rumination manipulation in the experimental task. All participants were first required to respond to the emotional n-back (Pe et al., 2013a), and then the anger-recall task. Both tasks were programmed in E-prime (Ray et al., 2008). Each session lasted for a maximum of 60 min. This study was approved by the local ethics committee of the Faculty of Psychology and Educational Sciences, KU Leuven, Belgium. Written informed consent was obtained from all participants.

#### Materials

##### Updating task

The updating task was identical to the task in Study 1.

##### Anger recall task

The anger recall task was adapted from Ray et al. (2008). Participants began the task by rating their baseline anger (angry, irritated, furious), other negative emotions (NE; guilty, sad, anxious), and positive emotions (PE; happy, relaxed, hopeful, and loving) on a scale from 0 (not at all) to 6 (very much) (baseline phase). They were then instructed to identify their most recent unresolved angering event. Once they had identified the event, they were asked to press the “spacebar.” After, they were asked to recall the event as if it were happening right now for 1 min (recall phase). Following the recall phase, participants were instructed to again rate their current feelings of anger, NE, and PE. Next, participants were instructed to either ruminate on (*n* = 46) or reappraise (*n* = 47) the angering event (emotion regulation phase). In this phase, participants were again asked to think about the angering event for 1 min. In the rumination condition, they were asked to “think about the event from your own perspective, and turn it over and over in your mind. Focus on those things that initially made you feel and respond the way that you did” (Ray et al., 2008). To help participants ruminate about the angering event, we added guide questions on the instruction screen (e.g., Why were you angry? What made you feel that way?). In the reappraisal condition, participants were instructed to “think about the angering event from a different perspective from the one you used earlier. For example, you might try to see this event from the perspective of an impartial observer” (Ray et al., 2008). To help participants reappraise the angering event, we added guide questions on the instruction screen (e.g., How would this person see the event? What positive outcomes of the event would this person think of?). After, participants were again asked to rate their current levels of anger, NE, and PE. This was then followed by a “rest” phase, in which participants were shown a neutral image (a ball of colored yarn) for 30 s, with instructions to “wait for the next screen.” After, they were again asked to report their current levels of emotions. The last two phases (emotion regulation phase and rest phase) were completed two more times, with a total of three iterations. Finally, participants did a distracting task, which was composed of three blocks. They reported their emotions after each of these blocks. At the end of the task, participants were also asked to rate on a scale from 0 (not at all) to 6 (very much) two questions probing about the perspective, in which they had viewed the angering event: How much did you see the angering event from your own perspective? (self-perspective), and how much did you see the angering event from a third-person perspective? (other-perspective).

Overall, the anger-recall task had a total of 11 emotion measurement occasions. For each of the measurement occasions, we averaged ratings from the three anger items to form an anger scale (between-subject reliability = 0.96), three negative emotion items to form an NE scale (between-subject reliability = 0.96), and four positive emotion items to form a PE scale (between-subject reliability = 0.97).

However, to respond to the goal of the present study, we only analyzed data from the first four measurement occasions, which allowed us to have a purer measure of emotion reactivity to and emotion recovery from the angering event.

#### Statistical model

We used the same model as in Study 1, however this time, we had four phases: Baseline, recall, emotion regulation, and rest. Similarly, measurement occasions during the different phases were recoded into numerical form: Measurement occasion during baseline was coded as 1, the measurement occasion right after the recall of the angering event was coded as 2, the measurement occasion right after the emotion regulation manipulation was coded as 3, whereas the measurement occasion after the rest period was coded as 4. To create the time variable, the phases were recoded to −1, 0, 1, and 2, respectively.

To account for the emotion regulation manipulation that occurred in between the anger recall and the rest phases, we added the emotion regulation manipulation condition (ERmanip) as a Level 2 predictor:

$$\begin{array}{l} \delta_{0i}=\gamma_{00}+\gamma_{01}(updating_{i})\;+\;r_{0i}\\ \delta_{1i}=\gamma_{10}+\gamma_{11}(updating_{i})\;+\;r_{1i}\\ \delta_{2i}=\gamma_{20}+\gamma_{21}(ERmanip_{i})\;+\;\gamma_{21}(updating_{i})\;+\;r_{2i} \end{array}$$

This analyses was done three times, one for each emotion variable as a dependent variable (anger, NE, and PE).

In another set of analyses, we also included the interaction between emotion regulation condition and updating ability. Since this interaction was not significant in predicting any of the dependent variables, it was not included in the final model. In addition, we also analyzed our data with the ERmanip variable added to predict the δ_0*i*_ and δ_1*i*_ parameters. We also found no significant ERmanip group difference in the reactivity slope and emotional intensity when recalling the angering event.

### Results

Descriptive statistics of the variables used in Study 2 are presented in Table 3.

**Table 3:** **Descriptive statistics of the variables in Study 2 (*n* = 93)**.

		***M***	***SD***	**Actual range**	**Possible range**	**Correlations**											
						**1**	**2**	**3**	**4**	**5**	**6**	**7**	**8**	**9**	**10**	**11**	**12**
1	Emotional n-back	0.60	0.13	0.28−0.90	0.00–1.00	–											
2	Anger (baseline)	0.80	1.09	0.00–5.00	0.00–6.00	−0.03	–										
3	Anger (recall)	3.20	1.57	0.00–6.00	0.00–6.00	**0.26**	**0.21**	–									
4	Anger (ERManip)					0.19	0.18	**0.75**	–								
	Rumination	2.79	1.35	0.00–6.00	0.00–6.00												
	Reappraisal	2.50	1.58	0.00–6.00	0.00–6.00												
5	Anger (rest)					0.08	**0.28**	**0.68**	**0.83**	–							
	Rumination	2.11	1.45	0.00–6.00	0.00–6.00												
	Reappraisal	2.22	1.67	0.00–6.00	0.00–6.00												
6	NE (baseline)	0.90	1.16	0.00–6.00	0.00–6.00	0.08	**0.51**	0.11	0.10	0.18	–						
7	NE (recall)	1.49	1.28	0.00–5.67	0.00–6.00	0.03	**0.32**	**0.21**	**0.23**	**0.31**	**0.66**	–					
8	NE (ERManip)					−0.03	**0.23**	0.20	**0.27**	**0.29**	**0.55**	**0.83**	–				
	Rumination	1.43	1.18	0.00–4.33	0.00–6.00												
	Reappraisal	1.35	1.21	0.00–4.33	0.00–6.00												
9	NE (rest)					−0.04	**0.28**	**0.27**	**0.34**	**0.45**	**0.62**	**0.79**	**0.86**	–			
	Rumination	1.12	1.12	0.00–4.00	0.00–6.00												
	Reappraisal	1.24	1.32	0.00–4.33	0.00–6.00												
10	PE (baseline)	3.44	1.08	0.75–6.00	0.00–6.00	0.18	**−0.44**	0.01	−0.07	−0.10	**−0.34**	−0.18	−0.20	**−0.31**	–		
11	PE (recall)	2.18	1.27	0.00–5.75	0.00–6.00	−0.12	−0.18	**−0.54**	**−0.45**	**−0.39**	**−0.21**	−0.16	−0.20	**−0.26**	**0.49**	–	
12	PE (ERManip)					−0.15	−0.17	**−0.44**	**−0.50**	**−0.43**	−0.16	−0.16	**−0.23**	**−0.26**	**0.53**	**0.86**	–
	Rumination	2.43	1.21	0.00–4.50	0.00–6.00												
	Reappraisal	2.38	1.59	0.00–6.00	0.00–6.00												
13	PE (rest)					0.03	−0.16	**−0.29**	**−0.33**	**−0.38**	**−0.21**	−0.12	**−0.21**	**−0.29**	**0.62**	**0.81**	**0.87**
	Rumination	2.89	1.26	0.00–5.00	0.00–6.00												
	Reappraisal	2.65	1.52	0.00–6.00	0.00–6.00												

*ERManip, emotion regulation manipulation; Because of the ER manipulation that occurred during the ERManip phase, we present separate Means and SDs for the rumination (n = 46) and reappraisal (n = 47) groups in the ERManip and rest phases. Since emotion regulation manipulation did not significantly influence our main results, correlations are presented with combined data from these two groups. NE, negative emotions; PE, positive emotions; Figures in bold are significant at p < 0.05*.

#### Manipulation check

To examine whether the emotion regulation manipulation was effective, we compared the extent to which participants from the two groups (rumination vs. reappraisal) viewed the angering event from their own or from another person's perspective. We conducted two independent samples *t*-tests, with either the self-perspective or other-perspective as the dependent variable, and the emotion regulation manipulation condition as the independent variable. Results revealed that although the two groups did not significantly differ in the extent to which they viewed the event from their own perspective (self-perspective), *t*_(91)_ = 1.23, *p* = 0.223, *d* = 0.26, they significantly differed on how much they viewed the event from another person's perspective, *t*_(91)_ = −4.10, *p* < 0.001, *d* = −0.86. Participants who were instructed to reappraise (*M* = 3.36, *SD* = 1.63) viewed the event more from another person's perspective compared to those who were instructed to ruminate (*M* = 2.00, *SD* = 1.56).

In addition, we also examined whether participants from the two conditions differed in the extent to which their emotions changed right after they were instructed to regulate their emotions. To measure change in emotions, we created a difference score between participants' emotion scores after recalling the angering event and after the emotion regulation manipulation, e.g., *Anger*_*recall*_ − *Anger*_*ERmanip*_. Higher numbers imply greater decrease in emotions from the recall phase to the emotion regulation phase. As expected, participants in the reappraisal condition (*M* = 0.89, *SD* = 1.09) experienced a greater decrease in their anger emotions relative to those in the rumination condition (*M* = 0.20, *SD* = 0.96), *t*_(91)_ = −3.24, *p* = 0.002, *d* = −0.68. No group differences in emotions were found for NE, *t*_(91)_ = −1.07, *p* = 0.228, *d* = −0.22, and PE, *t*_(91)_ = 1.62, *p* = 0.108, *d* = 0.34. These results suggest that the participants complied with the emotion regulation instructions.

#### Reactivity and recovery to film clips

##### Emotion reactivity

Results showed that after recalling the angering event, participants, on average, experienced a significant increase in anger and in NE and a significant decrease in PE from their baseline levels. The significant change of their emotions from baseline to the emotion-eliciting event (i.e., recall of a recent angering event) suggests that participants, on average, exhibited emotion reactivity to recalling an angering event (see γ_10_ in Table 4).

**Table 4:** **Moderating effect of updating on emotion reactivity to and emotion recovery from recalling a personal angering event in Study 2**.

	**Anger**				**NE**				**PE**			
	**Coef**	***SE***	**95% CI**	***R*^2^**	**Coef**	***SE***	**95% CI**	***R*^2^**	**Coef**	***SE***	**95% CI**	***R*^2^**
**RECALL AN ANGERING EVENT**												
Intercept (γ_00_)	3.19^**^	0.15	(2.90, 3.48)		1.51^**^	0.13	(1.26, 1.76)		2.16^**^	0.13	(1.91, 2.41)	
Updating (γ_01_)	0.41^**^	0.14	(0.14, 0.68)	0.07	0.02	0.12	(−0.21, 0.26)	−0.01	−0.20	0.10	(−0.40, −0.00)	0.01
**REACTIVITY**												
Intercept (γ_10_)	2.39^**^	0.17	(2.06, 2.72)		0.61^**^	0.11	(0.39, 0.83)		−1.28^**^	0.12	(−1.52, −1.05)	
Updating (γ_11_)	0.44^**^	0.16	(0.13, 0.75)	0.08	−0.07	0.09	(−0.25, 0.11)	−0.01	−0.39^**^	0.11	(−0.61, −0.17)	0.13
**RECOVERY**												
Intercept (γ_20_)	−0.46^**^	0.08	(−0.62, −0.30)		−0.16^**^	0.05	(−0.26, −0.06)		0.26^**^	0.06	(0.14, 0.38)	
ERManip (γ_21_)	−0.12	0.12	(−0.36, 0.12)		0.00	0.07	(−0.14, 0.14)		0.07	0.08	(−0.09, 0.23)	
Updating (γ_22_)	−0.13^*^	0.06	(−0.25, −0.01)	0.07	−0.04	0.03	(−0.10, 0.02)	−0.00	0.09^*^	0.04	(0.01, 0.17)	0.10

*ERManip, emotion regulation manipulation; Similar to Table 2, we used R^2^, the proportion of reduction in variance, to compute for effect sizes. Since we were interested at the proportion of variance explained by adding the updating ability as a predictor, the base model (subscript with nopredictor; see Table 2) had emotion regulation condition as a predictor, while the comparison model (subscript with predictor; see Table 2) included updating ability as a predictor. We would again like to note here that the cautionary note from Table 2 is likewise applied to this Table*.

** p < 0.01;

* *p < 0.05*.

##### Emotion recovery

Results showed that following a short rest period after recalling the angering event, participants, on average, experienced a significant decrease in anger and NE, and a significant increase in PE. These results suggest that on average, participants reported emotion recovery from recalling the angering event (see γ_20_ in Table 4).

#### Updating and emotion reactivity and recovery

##### Emotion reactivity

**Figure 2:**
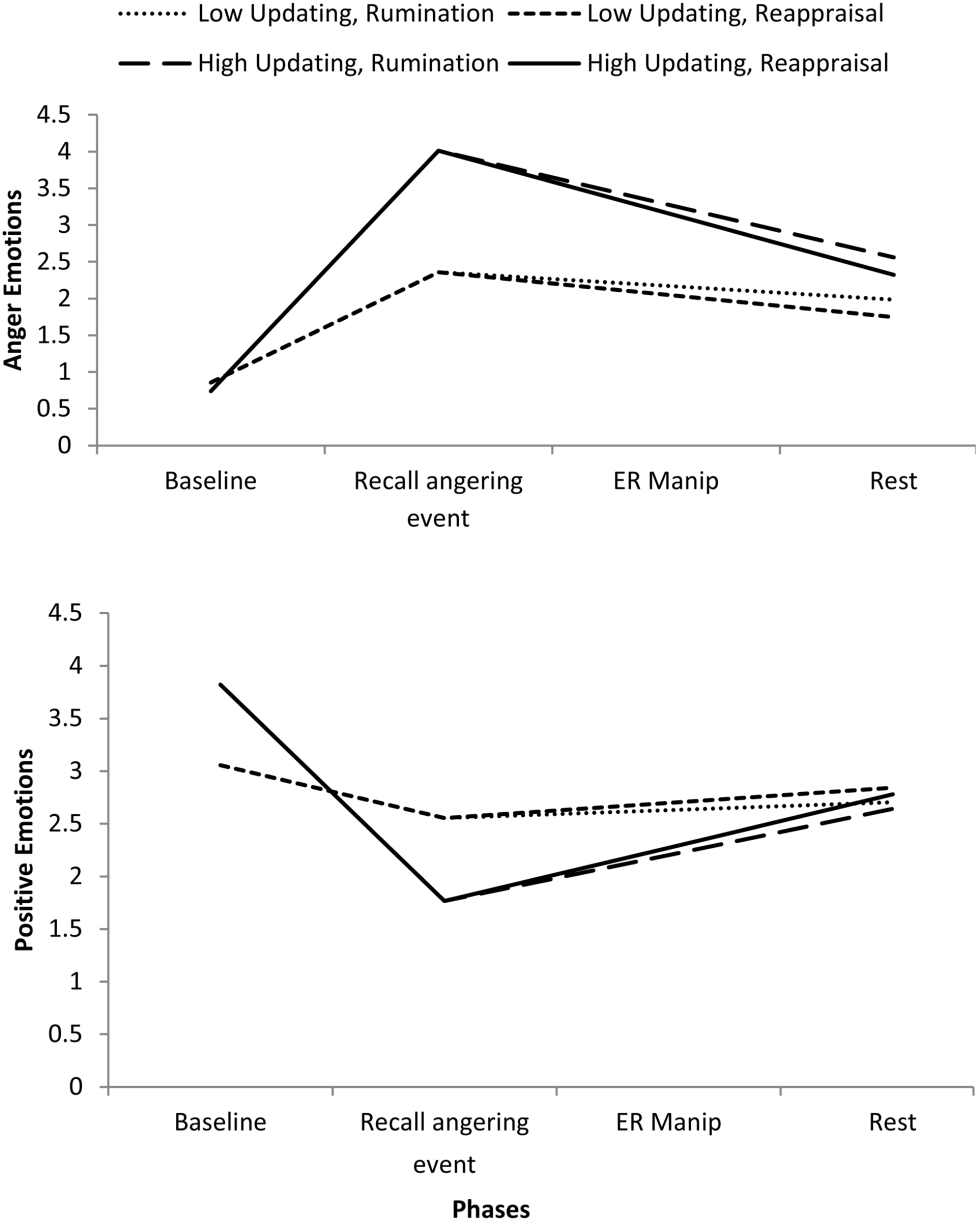
**Anger and positive emotions in Study 2 during the different phases of the anger recall task**. Low and high updating represent participants who scored 2SD below and above the sample average emotional updating ability, respectively.

Similar to the findings in Study 1, updating ability was significantly related to the strength of the reactivity of anger emotions in response to the angering event, such that the better the updating ability, the greater the increase in anger after recalling the angering event (see Table 4 and Figure 2). Simple slope analyses revealed that individuals with high updating ability (2*SD* above the mean) had a greater increase in anger (β = 3.27, *SE* = 0.37, *p* < 0.001) relative to those with low updating ability (2*SD* below the mean; β = 1.50, *SE* = 0.36, *p* < 0.001).

Interestingly, updating ability was also related to the strength of PE reactivity: The better the updating ability, the larger the decrease in PE after recalling the angering event (see Table 4 and Figure 2). Simple slope analyses revealed that individuals with high updating ability (2*SD* above the mean) had a significant decrease in PE (β = −2.06, *SE* = 0.28, *p* < 0.001) relative to those with low updating ability (2*SD* below the mean; β = −0.50, *SE* = 0.23, *p* = 0.03).

No significant relations were found between updating ability and NE reactivity (see Table 4 for full Results).

##### Emotion recovery

As expected, updating ability was significantly related to emotion recovery from recalling the angering event. Participants with better updating ability experienced a greater decrease in their anger and a greater increase in their PE after the rest period following the recall of an angering event (see Table 4 and Figure 2). Simple slope analyses revealed that individuals with high updating ability (2*SD* above the mean) had a significant decrease in anger (β = −0.73, *SE* = 0.17, *p* < 0.001), and a significant increase in PE (β = 0.44, *SE* = 0.13, *p* < 0.001), whereas this was not the case for individuals with low updating ability (2*SD* below the mean). Individuals with low updating ability did not have a significant decrease in anger (β = −0.19, *SE* = 0.11, *p* = 0.08), or a significant increase in PE (β = 0.08, *SE* = 0.07, *p* = 0.28) after the rest phase.

Again, no association was found between updating ability and NE recovery. Moreover, emotion regulation manipulation condition was not associated with the strength of the recovery (see Table 4 for full Results).

##### Other analyses

We also ran correlations to examine whether updating ability would be associated with levels of emotion (anger, NE, and PE) during the baseline and rest phases. Results showed no significant correlations between updating ability and emotion levels during baseline or rest (see Table 3).

#### Discussion

The findings in Study 2 replicated those from Study 1: Participants with better updating ability also experienced greater reactivity to and facilitated recovery. That is, although participants with better updating ability experienced greater reactivity in response to the angering event, they were also able to successfully recover from it. However, this was not the case for participants with low updating ability. Although participants with low updating ability experienced reactivity to the angering event (although this reactivity was blunted), they were not able to successfully recover from it.

In addition, we found that the use of putatively adaptive (reappraisal) vs. maladaptive (rumination) emotion regulation strategy did not have an influence on this relationship. Participants with high updating ability experienced successful recovery from recalling a recent angering event regardless of whether they ruminated or reappraised about this event. This result coincides with our previous work, which demonstrated that compared to individuals with low updating ability, those with high updating ability tend to experience greater emotion recovery when rumination or reappraisal was used to regulate their emotions (Pe et al., 2013b).

## General discussion

The findings of the present studies demonstrated that people's updating ability is related to their emotion reactivity to and recovery from negative emotion-eliciting stimuli. Specifically, individuals with high updating ability had greater emotion reactivity in response to watching negative films (Study 1), and recalling a recent angering event (Study 2). Notably, however, participants with high updating ability were also able to quickly or successfully recover; that is, they were able to bring their intense emotional responses back to baseline quickly (Study 1) and successfully (Study 2) once these negative events have passed. Moreover, the relationship between updating and emotion recovery was independent of use of emotion regulation strategies that are typically considered maladaptive (rumination) and adaptive (reappraisal) (Study 2).

Five features of this work limit the conclusions we draw about the relationship between updating emotional information in WM and emotion reactivity and recovery. First, the current study remains correlational; thus, we cannot make any conclusions regarding the directionality of our results. Does updating ability lead to greater emotion reactivity and facilitated recovery, or does greater emotion reactivity and facilitated recovery lead to improved updating ability? Future research can respond to this limitation by training participants to improve their updating ability and testing whether improved updating ability would also enhance their emotion reactivity and recovery. Although there are indications regarding the potential of training programs to improve executive control, of which updating ability is a component (Schweizer et al., 2013), researchers continue to question the validity of these findings (see Shipstead et al., 2012). Examining the relationship between updating and emotion reactivity and recovery in a longitudinal study is another possible, although less ideal, way of investigating the directionality of these findings. Although it does not determine causal effects, it can give a “snapshot” of the possible direction of the relationship (i.e., an event that occurs at Time 2 will never precede the event at Time 1).

Second, the current study cannot make any conclusions regarding the role of updating specifically emotional information in emotion reactivity and recovery. Although the current study used an emotional version of the n-back task, without a neutral n-back, we cannot ascertain that emotional updating adds predictive value above and beyond non-emotional updating. This would be an important hypothesis to test in the future. For now, what is clear, however, is that updating information in WM is related to emotion reactivity to and recovery from negative stimuli.

Third, whether emotion reactivity and recovery is specific to the executive function of updating cannot be answered in this study. Previous studies have demonstrated that both inhibition and switching are also implicated in emotion-regulation, particularly in the down-regulation of negative emotions (Joormann and Gotlib, 2008; De Lissnyder et al., 2012; Malooly et al., 2013). Researchers interested in disentangling the importance of each executive process on emotion responding should consider using various tasks that tap into the different executive functions, and simultaneously use performances on these tasks as predictors of emotion responding (e.g., McRae et al., 2012).

Fourth, the current study relies on self-report data to determine emotion reactivity and recovery. This kind of measurement is prone to demand characteristics; participants responses may be influenced by their desire to respond in a more socially desirable manner (Furnham, 1986). Future research could also include other potentially more objective measures, like psychophysiological or neural indicators to measure emotion reactivity and recovery.

Lastly, in Study 2, we cannot make strong conclusions regarding the recovery slopes of the low updating individuals. Since low (vs. high) updating individuals had blunted anger and PE reactivity in response to the angering event (e.g., their level of anger during the anger recall was significantly lower relative to the high updating individuals), their recovery slopes would never reach the same magnitude as the high updating individuals. Therefore, because of the differences in their initial reactivity scores, the conclusion that individuals with low updating ability have impaired recovery cannot be strongly asserted. However, what is interesting in Study 2 is that relative to their emotion levels at baseline, low updating individuals experienced an increase (decrease) in their anger (PE) scores in response to the angering event (i.e., their anger and PE reactivity slopes were significant); we cannot, however, ascertain whether they successfully recovered from the angering event (i.e., their anger and PE recovery slopes were not significant). This is not the case for individuals with high updating ability. They demonstrated both emotion reactivity to and emotion recovery from the angering event. This suggests that in Study 2, although we cannot make strong conclusions regarding the recovery of individuals with low updating ability, we can infer that individuals with high updating ability were able to successfully recover from the angering event.

This study contributes to the literature in several ways. First, we provide empirical evidence that updating, an executive control process, is involved in emotion reactivity and recovery. Previous research has already implicated affective updating and WM (in general) as an underlying mechanism partly responsible for successful down-regulation of negative emotions (Schmeichel et al., 2008; Schmeichel and Demaree, 2010; McRae et al., 2012; Pe et al., 2013b). It seems reasonable that the ability to update information in WM would also be implicated in emotion reactivity and recovery (at least in response to negative stimuli) since this executive process is specifically involved in both coding new information and changing the contents of WM to accommodate the new information (Morris and Jones, 1990; Miyake et al., 2000). Since our emotions are, more often than not, a reflection of what is currently in our WM (or focus of attention), the ability to update emotional information in WM allows us to focus on, and therefore appropriately emotionally respond to the new event (e.g., negative event phase or rest phase). The current article, combined with previous studies, indicates the significance of updating information in WM on emotion responding.

Second, we found that updating ability is significantly related to emotion reactivity and recovery in two different contexts: standardized objective negative stimuli, in the form of negative film-clips (Study 1), and a more personally relevant event, in the form of a recent unresolved angering event (Study 2). These converging findings from two studies with different methodologies give us confidence regarding the possible critical role of updating ability in emotion reactivity and recovery.

Third, in studying emotional responding, we highlight the importance of studying *both* emotion reactivity and recovery since this would give researchers a more complete picture of the temporal unfolding of an emotional response (Koole, 2009). This is particularly relevant for clinical disorders that are heavily influenced by emotion dysregulation, such as depression. For example, depressed individuals are characterized by blunted emotion reactivity and impaired emotion recovery (Bylsma et al., 2008; Aldao et al., 2010), which may partly explain why their emotions tend to be more resistant to change in daily life (Kuppens et al., 2010; Pe et al., 2015). In the current study, we have demonstrated that individuals with high updating ability exhibited both increased reactivity and facilitated recovery. This is informative because it implies individuals with high updating ability demonstrate a more flexible and adaptive emotion responding: not only are they able to appropriately emotionally respond to negative events, but they are also able to quickly and successfully recover from them once the events have passed.

Finally, our findings indicate that the association between updating and emotion recovery is independent of emotion regulation use, at least in the case of rumination and reappraisal. This coincides with our previous result, which showed that participants with high updating ability tend to be more efficacious at regulating their emotions (Pe et al., 2013b). In other words, individuals with high updating ability experienced more emotion recovery regardless of the emotion regulation strategy they used. This is interesting and may imply that since individuals with high updating ability are effective at regulating their emotions, this allows them to freely experience their negative emotions, such as anger, when a negative event is encountered (thus, showing heightened emotion reactivity). In contrast, individuals with low updating ability may have more difficulties down-regulating their emotions after a negative event is encountered, which may lead them to avoid a heightened emotion reaction when a negative event is encountered (thus, showing blunted emotion reactivity). Our findings indicate that individuals with low updating ability may on the one hand blunt a heightened emotion reaction in a controlled laboratory setting in which the emotional events are relatively simple and expected. Problems may arise however when they are exposed to real-world emotional events, which are often unexpected and more complicated; they have less opportunity to prepare for their initial emotional response, and/or there are more factors that are also influencing their emotional response. Once their initial emotion response to the emotional event reaches their critical point—a point in which they have an emotional response that they cannot fully recover from—then they will show impaired recovery and higher levels of negative emotions. Note that a similar mechanism may help to explain why the emotion reactivity of clinically depressed individuals differ when it is measured in response to negative stimuli in the laboratory (blunted reactivity) or negative events in real-life (heightened reactivity) (Bylsma et al., 2011); they are still able to manage their initial emotion responses in the laboratory, but not in real-life. Of course, this postulation is speculative and would require further research to support it.

In conclusion, what stands out in this work is that in response to negative emotion-eliciting stimuli, individuals with high updating ability have not only increased emotion reactivity, but also facilitated emotion recovery. These findings highlight the importance of considering updating ability as one critical component in emotion responding. Therefore, the next time another driver cuts you off, know that how upset you feel during and after that encounter may depend on your updating ability.

### Conflict of interest statement

The authors declare that the research was conducted in the absence of any commercial or financial relationships that could be construed as a potential conflict of interest.
